# Efficiency of Natural Deep Eutectic Solvents to Extract Phenolic Compounds from *Agrimonia eupatoria*: Experimental Study and In Silico Modelling

**DOI:** 10.3390/plants11182346

**Published:** 2022-09-08

**Authors:** Mila Lazović, Ilija Cvijetić, Milica Jankov, Dušanka Milojković-Opsenica, Jelena Trifković, Petar Ristivojević

**Affiliations:** 1 Innovation Centre of Faculty of Chemistry Ltd., Studentski Trg 12-16, 11158 Belgrade, Serbia; 2 University of Belgrade–Faculty of Chemistry, Studentski Trg 12-16, 11158 Belgrade, Serbia

**Keywords:** natural deep eutectic solvents, heating extraction method, UHPLC-DAD-MS/MS, conductor-like screening model for realistic solvation, flavonol glycosides

## Abstract

To replace common organic solvents that present inherent toxicity and have high volatility and to improve the extraction efficiency, a range of natural deep eutectic solvents (NADESs) were evaluated for the extraction of phenolic compounds from *Agrimonia eupatoria*. Screening of NADES efficiency was carried out based on the total phenolic and flavonoid content and radical-scavenging activity, determined by spectrophotometry, as well as phenolic compounds quantified, obtained using ultra-high-performance liquid chromatography with a diode array detector and a triple-quadrupole mass spectrometer. Increased extraction efficiency when compared with organic solvent was achieved using NADES mixtures choline chloride (ChCl):urea 1:2 and choline chloride:glycerol 1:1. Flavonol glycosides were the most abundant compounds in all extracts. The COSMO-RS model provided insights into the most important intermolecular interactions that drive the extraction process. Moreover, it could explain the extraction efficiency of flavonol glycosides using ChCl:glycerol NADES. The current article offers experimental evidence and mechanistic insights for the selection of optimal NADES to extract bioactive components from *Agrimonia eupatoria*.

## 1. Introduction

*Agrimonia eupatoria* is a medicinal plant from the Rosaceae family and it is widespread in Central and Northern Europe but can also be found in Asia and North America. *A. eupatoria* is traditionally used in Balkan countries for its therapeutical effects since it has anti-inflammatory, antibacterial, and hemostatic properties. It has been used for the treatment of digestive tract diseases against gall bladder stones, colic, and urinary disorders and for mucus membrane and skin ulcers. Moreover, *A. eupatoria* is used for the treatment of metabolic disorders such as diabetes, as well as in the protection of the cardiovascular and respiratory system [1,2,3]. *A. eupatoria* is a source of various phytochemicals which may be responsible for its beneficial effects. Phenolics, including tannins (2%), flavonoids (1.2–1.4%), and phenolic acids (2.26%), have been reported as a major group of phytochemicals in the dried plant [2]. A comprehensive study of the phenolic compounds found in *A. eupatoria* was carried out by Granica and associates and resulted in the identification of 24 phenolic compounds including phenolic acids, flavan-3-ol derivatives, ellagitannin, and flavonoids [4]. Moreover, they quantified 14 phenolics and showed that the tannins were the major group present in the *A. eupatoria* with a concentration of 6.3–10.9 mg/g, and the most abundant tannin was agrimoniin with an amount of 2.6–5.4 mg/g. Flavonoids were the second major group (8.2–10.9 mg/g) and 7-O glucuronides of luteolin and apigenin were the most dominant flavonoids although quercetin derivatives were present as well. Phenolic acids were present in the range of 0.6–0.9 mg/g. Another study of phenolic compounds in two species of genus *Agromenia L.* suggested that flavonoids vitexin and isovitexin (apigenin C-glycosides) could be chemotaxonomic markers of *A. eupatoria* [5].

Different solvents have been studied for the extraction of phenolics from *A. eupatoria* and methanol, ethyl acetate, and hydroalcoholic extract (tincture) were most often used [1,6,7]. Organic solvents have great extraction efficiency; however, the obtained extracts are often unsafe for human consumption due to solvent toxicity. On the contrary, tinctures are safe for oral use, but the extraction yields are lower compared with organic solvents. Except for their toxicity, organic solvents have a negative impact on the environment since they are thermally unstable, flammable, corrosive, and poorly biodegradable. Therefore, one of the aims of green chemistry is to eliminate or to significantly reduce their usage. Different strategies are explored to achieve these goals such as solventless extraction, supercritical fluid extraction, and application of ionic liquids as medium for extraction [8,9,10]. In recent years, there has been growing interest in deep eutectic solvents (DESs) which is a new class of ionic liquids (ILs) that are environmentally friendly and safe for use.

DESs is a eutectic mixture of two or more components which are hydrogen bond acceptors (HBA) and hydrogen bond donors (HBDs). The constituents interact through hydrogen bonding giving a liquid mixture that has a lower melting point than individual components, when combined at the proper molar ratio. Some DESs have been developed from the combination of primary plant metabolites, such as sugar alcohols, sugars, amino acids, organic acids, and amines, and they are named “natural deep eutectic solvents” (NADESs). The natural origin of NADESs makes them nontoxic, biodegradable, and environmentally friendly solvents. A great advantage of NADESs is their adjustable polarity that can be modified by changing the combination and ratios of HBA and HBD which gives them the ability to dissolve a wide range of bioactive compounds. NADESs have high viscosity because of the extensive hydrogen-bonding network between the compounds that makes them difficult to work with. Viscosity can be a drawback in the application of NADESs as medium for extraction due to reduced mass transfer which results with lower extraction yield, but viscosity can be decreased by adding water or increasing temperature. However, the extension of dilution with water will cause a loss of existing hydrogen bonds, and consequently, NADES structure [11]. Numerous combinations of HBA and HBD and the possibility of tailoring their physicochemical properties makes NADESs designer solvents [12,13,14,15].

Some authors suggest that NADESs could be present in living organisms in the form of a dynamic sheet around membranes which is made from positively charged choline from membrane lipids bonded with carboxylic acids, amino acids, or sugars [16]. In this dynamic sheet of NADES, bioactive compounds such as antioxidants could be concentrated, which protects membranes from oxidation and damage. The presence of NADES in cells can explain the concentration of compounds such as flavonoids and anthocyanins that is much higher than their solubility in water. NADES represent a great green alternative to organic solvents, and in recent years, numerous studies have explored the extraction of phenolic compounds using NADESs from different natural sources [17,18].

The choice of optimum solvent for the extraction of a compound of interest is a difficult task since the properties of NADES depend on the nature of HBD and HBA. The most widely used method for the rational selection of NADES is COSMO-RS (conductor-like screening model for real solvents). This method is based on σ-profiles, which represent surface charge densities obtained from quantum chemical calculations. The method converts σ-profiles into chemical potentials that allow the calculation of macroscopic properties such as thermodynamic solubility using statistical thermodynamics. Analogously to the “like dissolves like” principle, high solubility of a solute is expected in solvents having similar σ-profiles. COSMO-RS has shown good predictive ability for screening the optimum NADES for liquid–liquid and solid–liquid extractions [19,20,21,22,23,24].

The aim of this study was to build an efficient environmentally friendly method for the extraction of flavonol glycosides from *A. eupatoria* using NADES. This goal will be achieved through: (a) an investigation of extraction efficiency of nine applied NADESs based on the total phenolic content (TPC) and total flavonoid content (TFC) of extracts, as well as radical-scavenging activity (RSA); (b) quantification of individual phenolic compounds using ultra-high-performance liquid chromatography with a diode array detector and a triple-quadrupole mass spectrometer (UHPLC-DAD-MS/MS); (c) application of the COSMO-RS model to understand the molecular interactions between solute and solvent; (d) correlation of experimental and theoretical data to check the applicability of COSMO-RS for the prescreening of extractant.

## 2. Results and Discussion

### 2.1. Screening of NADES for Extraction of A. eupatoria Bioactives

Depending on the physicochemical properties of the target compounds and plant material characteristics, a large variety of NADESs could be selected as optimal solvents. The screening of the extraction solvent is, therefore, an initial step in the study of extraction of bioactive compounds. Nine NADES mixtures prepared from 11 primary plant metabolites (Table 1) were tested for extraction efficiency of phenolic compounds from aerial parts of *A. eupatoria*. Compounds used for synthesis of NADES were selected to target the extraction of flavonoids, the second major group of phenolics in *A. eupatoria*. Choline chloride and L-proline are commonly used as HBDs, and polyhydroxy alcohols and carboxylic acids as HBA components of NADES, in the case of flavonoid extraction [17]. The viscosity of NADESs is a major drawback that can limit their use as extraction solvents due to the lower mass transport efficiency. The viscosity is influenced by temperature and the water content. The increase in the temperature decreases the viscosity of the mixtures. It was shown that viscosity noticeably decreased with the change in temperature from room to 50 °C [12,25]. However, it should be also considered that some phytochemicals are sensitive to elevated temperatures [17]. Considering the above-mentioned facts, the extraction temperature was set to be 50 °C. Apart from temperature, water greatly affects the viscosity of NADES, and to evaluate its effect, the content of added water (ranging from 20% to 50%, *w*/*w*) was optimized (Section 3). The extraction process was not improved as the water content in NADES increased, i.e., any regular trend among the data could not be observed with the addition of water (Supplementary Materials, Tables S1 and S2). Therefore, in order to avoid the change in interactions between the target compounds and NADES caused by the higher content of water [11], it was set to be 20% (*w*/*w*). All extraction parameters were held constant in order to obtain information about the influence of NADES type on extraction yield. Methanol, as conventional solvent, was used as contrastive solvent.

**Figure 1:**
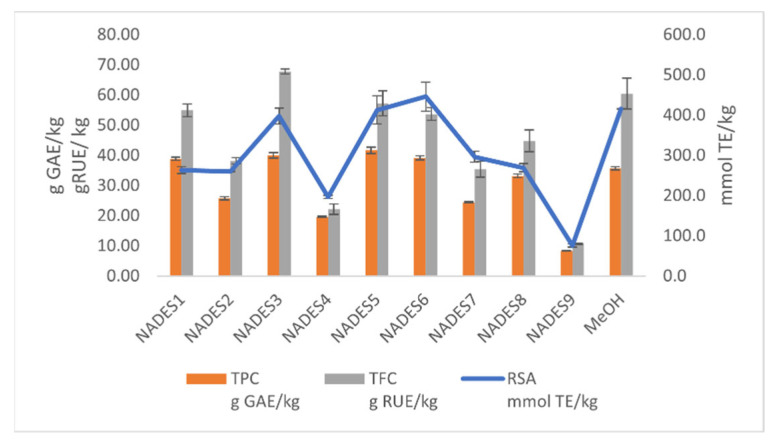
TPC, TFC, and RSA of *A. eupatoria* obtained using NADES and methanol.

The extraction efficiency of the applied NADESs was evaluated according to the total phenolic content (TPC) and total flavonoid content (TFC) of the extracts, as well as radical-scavenging activity (RSA) (Figure 1 and Table S3). TPC, TFC, and RSA values of *A. eupatoria* NADES extracts were in the range of 8.32 g GAE/kg to 41.65 g GAE/kg, 10.69 g RUE/kg to 67.79 g RUE/kg, and 75.6 mmol TE/kg to 445.6 mmol TE/kg, respectively. The extraction efficiency of target compounds differed mutually and depended on the NADES. Several applied solvents had equivalent or even higher extraction efficiency compared to methanol, indicating their possibility to be green alternatives to organic solvents. Among all the studied solvents, the highest TPC values were found for NADES5 (choline chloride:glycerol 1:1) followed by NADES3 (choline chloride:urea 1:2), NADES6 (glycerol:urea 1:1), and NADES1 (L-proline:malic acid 1:1). All listed NADES extracts had higher TPC values compared to methanol. When the TFC values of the tested solutions were compared, only extract NADES3 (choline chloride:urea 1:2) had a higher value than methanol, while NADES5 (choline chloride:glycerol 1:1), NADES1 (L-proline:maleic acid 1:1), and NADES6 (glycerol:urea 1:1) had similar values. Different TPC and TFC values of the tested extracts (NADES2-5 or NADES6-9) which had the same HBA (choline chloride or glycerol) and various HBDs (urea, glycerol, tartaric acid, succinic acid or urea, lactic acid, and L-ascorbic acid) showed that changing one NADES component strongly influenced the extraction. Similarly, changing the ratio between the HBD and HBA, in the case of glycerol:urea (NADES5 and NADES6), also led to notably different TPC and TFC values. An increase in the glycerol:urea ratio from 1:1 to 2:1 reduced the extraction efficiency probably due to the changed solvent viscosity. Generally, the type of NADES and ratio among its components influenced the extraction efficiency of phenolic compounds in *A. eupatoria,* indicating the importance of hydrogen bonding between NADES and the target components. Namely, changing the solvent viscosity and the number of hydrogen bonds contribute to the breakage of the plant cell wall and the dissolution of target components [26].

NADES5 (choline chloride:glycerol 1:1), NADES6 (glycerol:urea 1:1), and NADES3 (choline chloride:urea 1:2) extracts exhibited higher or similar ability to act as free radical scavengers as methanol (Figure 1 and Table S3). The lowest antioxidant activity (as well as TPC and TFC values) was recorded for NADES9 (glycerol:L-ascorbic acid 1:1) extracts. Good Pearson’s correlation was observed between TPC and RSA values (*r* = 0.876), suggesting that phenolics were the main compounds responsible for the antioxidant activity of *A. eupatoria* extracts.

In agreement with the results for TPC, TFC, and RSA, mixtures NADES5 (choline chloride:glycerol 1:1), NADES6 (glycerol:urea 1:1), and NADES3 (choline chloride:urea 1:2) could be marked as the most promising solvents for the extraction of phenolic compounds from *A. eupatoria.*

### 2.2. Quantification of Individual Phenolic Compounds

In order to further evaluate the efficiency of NADESs to extract phenolic compounds from *A. eupatoria,* UHPLC-DAD-MS/MS analysis was performed. Eighteen phenolic compounds were determined in NADES extracts (Table 2). The most abundant group of flavonoids was flavonol glycosides, among them 3-O-glycosides of quercetin: quercitrin, isoquercitrin, and rutin, and 3-O-glycoside of kaempferol–astragalin.

Quercitrin (quercetin 3-O-rhamnoside) was the predominant compound present in *A. eupatoria* found in the highest concentrations in all solvents in the range of 304.91 mg/kg to 936.37 mg/kg. Four NADES mixtures extracted a higher amount of quercitrin compared to methanol, NADES5 (choline chloride:glycerol 1:1), NADES3 (choline chloride:urea 1:2), NADES6 (glycerol:urea 1:1), and NADES8 (glycerol:lactic acid 1:1). Isoquercitrin (quercetin 3-O-glucoside) was the second most abundant flavonoid in all solvents with concentrations between 189.48 and 612.03 mg/kg; NADES5 (choline chloride:glycerol 1:1), NADES3 (choline chloride:urea 1:2), and NADES6 (glycerol:urea 1:1) extracted a higher amount than methanol. Astragalin (kaempferol 3-O-glucoside) was found in the range of 92.79 mg/kg to 401.29 mg/kg and it was the third most abundant flavonoid quantified in *A. eupatoria*. Six NADESs proved to be more efficient than methanol. NADES5 (choline chloride:glycerol 1:1) extracted a 1.9 times higher amount of astragalin than methanol. Rutin (quercetin 3-O-rhamnoglucoside) was extracted in the range of 32.50 mg/kg to 198.79 mg/kg and NADES5 (choline chloride:glycerol 1:1) showed a 2.1 times higher concentration of rutin compared to methanol. The flavonols quercetin and kaempferol were best extracted with NADES1 (L-proline:maleic acid 1:1) and NADES2 (choline chloride:tartaric acid 1:1), where their content was 5.4 and 4.9 times, i.e., 2.8 and 2.1, respectively, higher than in methanol. Experimental data showed that NADES5 (choline chloride:glycerol 1:1) had the greatest efficiency for the extraction of flavonol glycosides from *A. eupatoria* among all tested solvents, followed by choline chloride:urea.

Additionally, seven phenolic acids were quantified in NADES extracts and the most abundant were protocatechuic, syringic, chlorogenic, and p-hydroxybenzoic acids. Chlorogenic acid was extracted 5.5 and 3.6 times more by NADES3 (choline chloride:urea 1:2) and NADES5 (choline chloride:glycerol 1:1), respectively, than with methanol, while NADES1 (L-proline:maleic acid 1:1) extracted a 2.9 times higher amount of syringic acid compared with methanol.

The phenolic profiles of *A. eupatoria* (presented by eighteen quantified compounds), obtained by methanol and each NADES separately, were compared using a paired *t*-test (Table S4). Two extraction solvents were tested by applying both of them to the same set of phenolic compounds, which contained different amounts of analyte, by looking at the difference between each pair of results. Significantly higher amounts of phenolic compounds were extracted by NADES3 (choline chloride:urea 1:2) and NADES5 (choline chloride:glycerol 1:1) compared with methanol (*P*(*t* ≥ 2.22) = 0.04), making them more efficient extraction media than organic solvent. No statistically significant differences between methanol and other NADESs were observed, indicating equal extraction efficiency, but the low toxicity and biodegradability of NADES give them an advantage over conventional solvent (Table 2 and Table S4). Additionally, comparing the extraction efficiency of different NADES, significantly higher amounts of phenolic compounds were extracted by NADES3 (choline chloride:urea 1:2) and NADES5 (choline chloride:glycerol 1:1) compared to NADES6 (glycerol:urea 1:1) and NADES7 (glycerol:urea 2:1), while NADES1 (L-proline:maleic acid 1:1) extracted more phenolics compared to NADES2 (choline chloride:tartaric acid 1:1), NADES4 (choline chloride:succinic acid 1:1), and NADES9 (glycerol:L-ascorbic acid 1:1) (Table 2 and Table S4).

The UHPLC-MS results showed that a high content of phenolic compounds, particularly flavonol glycosides, could be obtained by NADES extraction.

### 2.3. COSMO-RS Predictions of Extraction Efficiency

**Figure 2:**
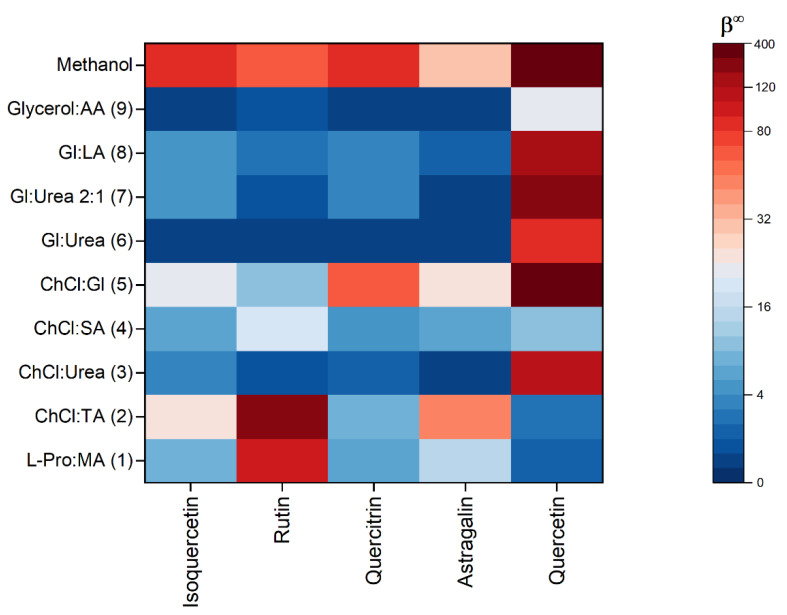
The solvent–solute affinity parameter β^∞^ for five solutes (x-axis) in ten solvents (y-axis). Higher values for β^∞^ imply better mutual affinity, i.e., higher extractability. The mol ratio of HBD and HBA in the NADES is 1:1 in all solvents except NADES7 (glycerol: urea 2:1). The abbreviations L-Pro, MA, ChCl, TA, SA, Gl, LA, and AA denote L-proline, maleic acid, choline chloride, tartaric acid, succinic acid, glycerol, lactic acid, and L-ascorbic acid, respectively.

To rationalize the extraction efficiency of NADES for flavonols and flavonol glycosides from *A. eupatoria*, we calculated activity coefficients at infinite dilution (γ^∞^) for the five most abundant compounds (isoquercetin, rutin, quercitrin, astragalin, and quercetin) in nine NADESs (Table 1), methanol, and water. Analogously to a previous study [19], γ^∞^ is converted to the parameter β^∞^ (β^∞^ = 1/γ^∞^) which is directly proportional to the affinity of solvent for a given solute. Figure 2 shows the predicted extraction efficiencies for five solutes in nine NADESs and methanol as a reference solvent. The β^∞^ values color-coded, and the exact values for all solvents along with the values for water solubility are given in Supplementary Materials, Table S5.

All NADES had higher affinity to phenolic compounds compared with pure water, and the relative affinity, calculated as the β^∞^ ratio in NADES and water, was from 100 to over 1,000,000 (Table S6). NADESs with choline chloride and organic acids show high solubilization enhancement toward rutin, and choline chloride:glycerol mixture enhanced quercitrin solubility compared with water. The results in Figure 2 show that NADES1 (L-proline:maleic acid 1:1) and NADES2 (choline chloride:tartaric acid 1:1) had higher affinity to rutin than methanol. NADES2 (choline chloride:tartaric acid 1:1) also had higher affinity to astragalin compared with methanol. We observed the highest affinity of the majority of NADESs toward quercetin. From the calculated log*P* values for five phenolics, we see that the quercetin was the most lipophilic compound in the set (Table S7). This result points to the importance of van der Waals and hydrophobic forces in the solubilization mechanism of studied compounds in NADES. To gain more insights into the mechanism of extraction, we calculated σ-profiles for all NADES and five compounds and studied their similarities.

**Figure 3:**
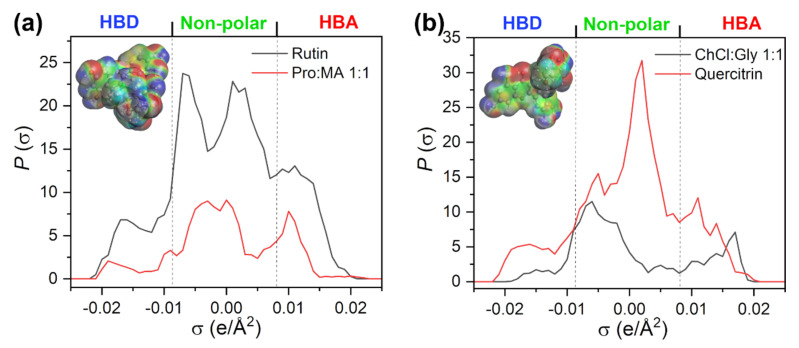
(**a**) The σ-profiles of a rutin and NADES1 (L-proline: maleic acid 1:1); (**b**) quercitrin and NADES5 (choline chloride: glycerol 1:1). The surface charge density of a solute is shown in the upper-left part of the graph.

The σ-profiles for NADES1 (L-proline:maleic acid 1:1) and rutin are shown in Figure 3a. σ-profiles represent histograms that give the probability of finding part of the surface with the surface charge density between σ and σ ± δσ. It represents the fingerprint feature of the molecule. The part of the profile with σ < −0.0084 e/Å^2^ represents the HBD region; between −0.0084 and 0.0084 e/Å^2^ is the region that maps nonpolar interactions; and the region above 0.0084 e/Å^2^ reflects the HBA affinity of a compound. Therefore, it is possible to compare the polarity of different molecules through simple analysis of the shape of σ-profile.

The HBD parts of both rutin and NADES1 (L-proline:maleic acid 1:1) are less pronounced than HBA, while nonpolar segments occupy the largest portion of their surface. The most likely hydrogen-bonding mechanism between rutin and NADES1 (L-proline:maleic acid 1:1) is via –OH groups of rutin as HBD and carbonyl O or amino N atoms from L-proline and maleic acid as HBA. It should be noted that all NADES constituents are modeled in their neutral forms, so it is likely that zwitterionic forms of amino acids and anionic forms of organic acids would interact more strongly with HBD groups of rutin. A previous study has shown that the solubility predictions for rutin in NADES consisting of amino acids and carboxylic acids require the accurate definition of protonation states of NADES constituents and the inclusion of all microstates in the COSMO-RS model [27].

Less polar solutes such as quercitrin interacted favorably with NADES5 (choline chloride:glycerol 1:1). From the corresponding σ-profiles (Figure 3b), it is clear that the lower polarity of a solute is reflected in the higher portion of surface charges in the nonpolar region. Moreover, the strong HBA peak around 0.018 e/Å^2^ is typical of choline chloride-based NADES so hydrogen bonding may occur between phenolic –OH groups of quercitrin and the cholinium cation as the HBA group.

The σ-profiles for rutin and NADES2 (choline chloride:tartaric acid 1:1) (Figure S1) reflect the stronger HBA ability of NADES which is favorable for the interaction with rutin –OH groups as HBDs. Isoquercetin, as the most polar compound in this series (log*P* = 1.877, Table S7), interacts favorably with choline chloride:glycerol mixture owing to the similar shape of the nonpolar region of the σ-profile and the good match between the HBA of NADES and the HBD part of flavonol glycoside (Figure S2).

### 2.4. Experimental Results vs. Theoretical Data

There are many possible combinations of HBA and HBD available for the extraction of bioactive compounds from natural products. However, it is a long-term process to experimentally evaluate their extraction performances individually for each prepared NADES. Additionally, the solubility of a chemical in a certain solvent could not be ambiguously determined by log*P* value or the rule ”like dissolves like”, as it could not give insight into the properties of the solute and solvent. In that sense, in order to better understand the molecular interactions between NADES and the target compounds, and to avoid time-consuming experiments, the solvent screening is usually performed using the COSMO-based thermodynamic model. The influence of HBA and HBD structures on the extraction efficiency are primary evaluated utilizing the COSMO model and then experimentally validated [19,28]. However, following such a sequence of evaluation, authors experimentally search for the best solvents among those previously theoretically selected.

On the contrary, among those reports in which COSMO-based analysis was used to provide a better understanding of the mutual solubilities during extractions which were previously experimentally performed, the results showed that it is difficult to make an unambiguous conclusion [17,26]. The simulated **γ^∞^** values showed an opposite trend compared to the experimental results, and the models performed better in the case of more polar solutes while generally failing to quantitatively predict experimental solubilities for solvent–solute combinations where nonpolar interactions play an important role [29]. Beside interactions between the solvent and solute modeled by COSMO-RS, physical parameters such as the viscosity and temperature largely affect the efficiency of solid–liquid extraction. The high viscosity of NADES slows down the mass transfer between the phases and prolongs the time needed to establish thermodynamic equilibrium. Moreover, the plant material matrix may interfere with the extraction mechanism. In this study, COSMO-RS predicted the high solubility of flavonol glycosides in NADES5 (choline chloride:glycerol 1:1). Since hydrophobic and van der Waals interaction are identified as important for the solubilization mechanism, higher extraction yields of flavonol glycosides from *A. eupatoria* might be obtained using hydrophobic DES [28].

## 3. Materials and Methods

### 3.1. Plant Material

The plant material (dried aerial part of the plant of *A. eupatoria*) was bought in a local shop in Belgrade, Serbia. The sample was milled for 5 min into a powder using a coffee grinder (Bosch Coffee Electric Grinder TSM6A017C) and stored in sealed containers at room temperature in the dark.

### 3.2. Chemicals and Reagents

Choline chloride, L-proline, succinic acid, methanol, sodium hydroxide, sodium carbonate, sodium-nitrite, 2,2-Diphenyl-1-picrylhydrazyl (DPPH), trolox, gallic acid, protocatechuic acid, syringic acid, chlorogenic acid, *p*-hydroxybenzoic acid, aesculetin, caffeic acid, isoorientin, rutin, vitexin, isoquercetin, *p*-coumaric acid, quercitrin, astragalin, rosmarinic acid, luteolin, quercetin, naringenin, kaempferol, and rutin were purchased from Sigma Aldrich Chemical Co. (St. Louis, MO, USA). Urea, ascorbic acid, and ethyl acetate were purchased from Betahem (Belgrade, Serbia). Lactic acid and tartaric acid were purchased from ICN Biomedicals Inc. (Aurora, OH, USA). Maleic acid and aluminum chloride were purchased from Acros Organics (Geel, Belgium). Folin–Ciocalteu reagent was purchased from Carlo Erba reagents (Milan, Italy). Glycerol was purchased from Zorka Pharma (Šabac, Serbia). All solvents and chemicals were analytical purity grade.

### 3.3. Preparation of NADES

The preparation of all NADES tested (Table 1) was carried out using the reflux method. Hydrogen bond acceptors (HBA), such as choline chloride, proline, and urea, and hydrogen bond donors (HBD), such as tartaric, succinic, maleic, lactic, L-ascorbic acid, and glycerol, were placed in Erlenmeyer flasks in a certain molar ratio (1:1 or 1:2). Mixtures were heated to 80 °C and constantly stirred for 30 min; then, water was added (20% *w*/*w*) and stirring continued for 30 min. To evaluate the effect of water content, different amounts of water were added (20–50%, *w*/*w*) to the three NADESs (choline chloride:tartaric acid (1:1), choline chloride:urea (1:2), and choline chloride:succinic acid (1:1)) during the optimization experiment.

### 3.4. Extraction with NADES and Conventional Solvent

Extraction of phenolic compounds was carried out using the reflux method. Briefly, 500 mg of plant material was placed in Erlenmeyer flasks and 5 mL of previously prepared NADES or methanol was added. The mixture was stirred for 45 min at a temperature of 50 °C. After extraction, samples were centrifuge ated at 10,000 rpm for 15 min. Removal of NADES from the extraction mixture and purification was carried out using solid-phase extraction (SPE) with octadecyl columns (Cartridge Bond Elut C18, Agilent Technologies, Santa Clara, CA, USA). Sorbents were conditioned with 5 mL methanol followed by 5 mL of water; then, 3 mL of sample was loaded on cartridge. An amount of 10 mL of water was used for washing of nonadsorbed compounds and NADES. Phenolic compounds were eluted with 1 mL of methanol and obtained extracts were stored at −20 °C in dark glass vials prior to analysis.

### 3.5. Determination of Total Phenolic Content (TPC)

Total phenolic content was determined for all NADES extracts using the Folin–Ciocalteu method. Briefly, 0.5 mL of the extracts and 0.5 mL ultrapure water were mixed with 2.5 mL of Folin–Ciocalteu reagent (10% *w*/*v*). The mixture was incubated for 5 min at room temperature and 2.0 mL of sodium carbonate (7.5%) was added. After incubation during 2 h at room temperature, absorbance was measured at 765 nm on a GBC UV-Visible Cintra 6 spectrophotometer. Gallic acid was used as standard in the range of 20−120 mg L^−1^ and a mixture of water and reagents was used as a blank. TPC values were expressed as gram of gallic acid equivalent (GAE) per kg of dry sample.

### 3.6. Determination of Total Flavonoid Content (TFC)

The total flavonoid content in extracts was determined using the aluminum chloride method and rutin as standard. Briefly, 0.3 mL of the extracts and 3.4 mL 30% *v*/*v* methanol were mixed with 0.15 mL of 0.5 M sodium nitrite and 0.15 mL of 0.3 M aluminum chloride. The mixture was incubated for 5 min at room temperature and 1.0 mL of 1 M sodium hydroxide was added and absorbance was measured at 506 nm. The concentration of standard rutin was in the range of 20−200 mg L^−1^, while 30% *v*/*v* methanol was used as a blank. TFC values were expressed as gram rutin equivalent (RUE) per kg of dry sample.

### 3.7. Determination of the Radical-Scavenging Activity (RSA)

Radical-scavenging activity was determined using DPPH radical solution. Briefly, 0.1 mL of the extracts was mixed with 4 mL of 79 μM methanol solution of DPPH. The mixture was incubated for 1 h at room temperature in the dark and absorbance was measured at 517 nm. Trolox was used as standard in the range of 100–600 μmol L^−1^. RSA was calculated as a percentage of DPPH discoloration using Equation (1), where *A*_DPPH_ is the absorbance of methanol solution of DPPH, and *A*_sample_ is the absorbance of samples. The results are expressed as millimoles of trolox equivalents per kg of dry sample.

(1) $$RSA\;\left(\%\right)=\frac{\left(A_{\mathrm{DPPH}}-A_{\mathrm{sample}}\right)}{A_{\mathrm{DPPH}}}\times 100$$

### 3.8. UHPLC-DAD-MS/MS

Quantification of phenolics in NADES extracts was performed using Dionex Ultimate 3000 UHPLC system (ThermoFisher Scientific, Waltham, MA, USA) configured with a diode array detector (DAD) and a triple quadrupole mass spectrometer (TSQ Quantum Access Max, ThermoFisher Scientific). An analytical Hypersil gold C18 column (50 × 2.1 mm) with 1.9 mm particle size (ThermoFisher Scientific) was used. The mobile phase consisted of 0.2% acetic acid in water (A) and LC-MS grade acetonitrile (B), was applied with a flow rate of 0.4 mL/min in the gradient elution as previously described [30]. The injection volume was 10 μL. The settings of a triple-quadrupole mass spectrometer (qqqMS), equipped with a heated electrospray ionization (HESI) source, were as follows: vaporizer temperature of 450 °C, spray voltage of 4000 V, sheet gas (N2) pressure of 50 AU, ion sweep gas pressure of 0 AU and auxiliary gas pressure of 20 AU, capillary temperature of 320 °C, and skimmer offset of 0 V. The mass spectrometer was operated in negative-ion mode, and the collision energy was 30 eV. Phenolic compounds were quantified using the external standard quantification procedure. Working standard solutions were prepared by dissolving the stock solution (1000 mg/L) of a pure compound to obtain a concentration of 100 mg/L. Dilution of the solution with mobile phase yielded working solutions at concentrations of 0.025, 0.050, 0.100, 0.250, 0.500, 0.750, and 1.000 mg/L. Calibration curves were obtained by plotting the peak areas of the standards against their concentration. Calibration curves revealed good linearity, with *R*^2^ values exceeding 0.99 (peak areas vs. concentration). The phenolic compounds were identified through direct comparison with commercial standards. The total amounts of each compound were evaluated by calculation of the peak areas.

### 3.9. COSMO-RS Analysis

The quantum chemical calculations of the activity coefficients at infinite dilution were carried out in Amsterdam density functional (ADF) COSMO-RS implementation in the ADF2022.101 program (https://www.scm.com/product/cosmo-rs/ (accessed on 16 July 2022) [31]. The ADFCRS-2018 database with over 2500 precomputed COSMO result files was used. For the compounds not listed in the database, the result files needed for the calculation of σ-profiles were calculated in the ADF package. All NADES constituents except ChCl were stored in the ADFCRS-2018 database. Although cholinium cation was on the list, we reconstructed the COSMO result file by modeling ChCl as an ionic pair. Other NADES constituents were modeled in their neutral form and the eutectic mixtures were modeled by combining HBA and HBD in a certain molar ratio. The .sdf files with the initial structures of five polyphenolics screened as solutes were downloaded from PubChem and their geometry was optimized using PM7 semiempirical quantum-mechanical model [32]. These geometries were reoptimized in ADF using the small core TZP basis set, the Becke–Perdew (GGA:BP86) functional, and the relativistic scalar ZORA method for gas-phase calculations. The surface charge densities and σ-profiles were calculated from the output files.

### 3.10. Statistical Analysis

Descriptive statistics and paired t-test were performed using Analysis ToolPak, Excel for Microsoft 365.

## 4. Conclusions

A range of NADESs consisting of HBAs—such as choline chloride, proline, and urea—and HBDs—such as tartaric, succinic, maleic, lactic, L-ascorbic acid, and glycerol—were used for the extraction of phenolic compounds from *A. eupatoria*. Increased extraction efficiency when compared with organic solvent was achieved using the mixtures NADES3 (choline chloride:urea 1:2) and NADES5 (choline chloride:glycerol 1:1). Flavonol glycosides were the most abundant compounds in all extracts. The extraction performance of the NADESs was additionally evaluated using COSMO-RS. Although the COSMO-RS methodology was useful for the determination of the extractability of flavonol glycosides in NADES5 (choline chloride:glycerol 1:1), indicating its promising possibilities in the screening of effective solvent, it was not able to offer good prediction for other applied solvents, although they proved to be effective. Therefore, we recommend that, apart from COSMO-RS results, an experiment should be carried out with a broader range of NADES mixtures. Additionally, higher extraction yields of flavonol glycosides from *A. eupatoria* might be obtained using hydrophobic DES.

## Figures and Tables

**Table 1:** The composition of the Natural Deep Eutectic Solvents (NADESs) used in the present study and their abbreviations.

Abbreviation	Component 1	Component 2	Molar Ratio	Water Content %
NADES1	L-proline	Maleic acid	1:1	20
NADES2	Choline chloride	Tartaric acid	1:1	20
NADES3	Choline chloride	Urea	1:2	20
NADES4	Choline chloride	Succinic acid	1:1	20
NADES5	Choline chloride	Glycerol	1:1	20
NADES6	Glycerol	Urea	1:1	20
NADES7	Glycerol	Urea	2:1	20
NADES8	Glycerol	Lactic acid	1:1	20
NADES9	Glycerol	L-Ascorbic acid	1:1	20

**Table 2:** Phenolic compounds quantified in *A. eupatoria* (mg/kg of dried sample) *.

	**Phenolic Compounds**								
	**Protocatechuic Acid**	**Syringic Acid**	**Chlorogenic Acid**	**p-Hydroxybenzoic Acid**	**Aesculetin**	**Caffeic Acid**	**Isoorientin**	**Rutin**	**Vitexin**
NADES1 ^a,m^	10.9 ± 1.6	47.7 ± 3.3	9.32 ± 0.23	23.6 ± 6.5	2.29 ± 0.22	2.68 ± 0.11	1.30 ± 0.08	103.0 ± 8.9	26.1 ± 2.4
NADES2 ^b,m^	10.10 ± 0.90	12.4 ± 1.8	11.3 ± 1.1	10.8 ± 1.8	1.92 ± 0.12	2.82 ± 0.18	0.75 ± 0.06	64.6 ± 5.5	15.9 ± 1.3
NADES3 ^a,b,d^	6.7 ± 1.2	15.2 ± 2.6	43.7 ± 3.2	14.1 ± 1.7	NF	3.49 ± 0.85	1.53 ± 0.14	147 ± 10	36.1 ± 2.6
NADES4 ^b,e,m^	3.97 ± 0.84	2.52 ± 0.88	9.21 ± 0.87	10.58 ± 0.95	1.39 ± 0.09	2.19 ± 0.10	1.01 ± 0.04	54.2 ± 2.4	14.6 ± 1.6
NADES5 ^a,b,d,h^	4.48 ± 0.88	5.69 ± 0.94	28.9 ± 2.5	20.6 ± 2.3	NF	1.32 ± 0.52	2.22 ± 0.20	198.8 ± 8.6	44.9 ± 2.5
NADES6 ^a,b,f,e,j,m^	3.29 ± 0.73	7.33 ± 0.75	4.88 ± 0.30	17.8 ± 1.3	0.57 ± 0.02	0.53 ± 0.07	1.89 ± 0.07	108.0 ± 9.2	24.0 ± 1.3
NADES7 ^a,b,g,e,k,j,m^	2.59 ± 0.94	9.13 ± 0.42	3.26 ± 0.19	12.4 ± 1.2	0.67 ± 0.03	0.54 ± 0.08	1.36 ± 0.12	100.2 ± 8.6	21.2 ± 1.6
NADES8 ^a,b,c,d,i,h,j,k,m^	8.8 ± 1.1	12.1 ± 1.7	11.5 ± 1.2	26.9 ± 2.4	3.47 ± 0.41	2.87 ± 0.24	0.82 ± 0.04	113.5 ± 9.3	23.8 ± 1.3
NADES9 ^c,d,e,h,j,k,m^	2.75 ± 0.79	4.05 ± 0.27	3.95 ± 0.23	8.45 ± 0.95	1.24 ± 0.27	0.94 ± 0.05	0.49 ± 0.02	32.5 ± 2.8	9.37 ± 0.93
MeOH ^m^	6.05 ± 0.88	16.4 ± 2.0	7.95 ± 0.98	17.8 ± 1.8	3.09 ± 0.35	3.98 ± 0.14	1.42 ± 0.11	92.9 ± 4.6	24.9 ± 2.2
	**Isoquercetin**	**p-Coumaric Acid**	**Quercitrin**	**Astragalin**	**Rosmarinic Acid**	**Luteolin**	**Quercetin**	**Naringenin**	**Kaempferol**
NADES1 ^a,m^	429.3 ± 8.6	4.93 ± 0.89	660 ± 11	242.0 ± 3.8	4.02 ± 0.27	0.85 ± 0.05	190.4 ± 8.4	1.66 ± 0.25	7.91 ± 0.34
NADES2 ^b,m^	316.3 ± 7.3	4.04 ± 0.23	460.9 ± 8.2	152.1 ± 2.9	1.92 ± 0.25	0.44 ± 0.05	170.5 ± 7.1	1.63 ± 0.80	5.92 ± 0.25
NADES3 ^a,b,d^	523.0 ± 8.9	9.4 ± 1.0	746.3 ± 9.6	314.5 ± 5.4	3.65 ± 0.65	0.94 ± 0.09	11.66 ± 0.90	1.96 ± 0.12	4.56 ± 0.08
NADES4 ^b,e,m^	338.1 ± 4.9	2.89 ± 0.12	469.8 ± 7.3	163.4 ± 1.3	1.27 ± 0.08	0.38 ± 0.02	21.0 ± 1.2	0.45 ± 0.08	2.26 ± 0.04
NADES5 ^a,b,d,h^	612.0 ± 9.2	6.15 ± 0.85	936 ± 12	401.3 ± 7.7	6.87 ± 0.86	1.26 ± 0.03	36.79 ± 0.85	1.33 ± 0.19	4.10 ± 0.11
NADES6 ^a,b,f,e,j,m^	500 ± 10	0.84 ± 0.03	707.5 ± 9.9	266.4 ± 4.6	3.24 ± 0.03	0.90 ± 0.12	31.04 ± 0.97	0.81 ± 0.04	4.59 ± 0.09
NADES7 ^a,b,g,e,k,j,m^	454 ± 10	0.39 ± 0.02	668.0 ± 7.6	254.4 ± 3.5	2.31 ± 0.02	0.86 ± 0.03	29.93 ± 0.92	0.89 ± 0.06	4.11 ± 0.05
NADES8 ^a,b,c,d,i,h,j,k,m^	432.3 ± 9.8	6.31 ± 0.12	702.0 ± 6.3	237.5 ± 3.0	3.20 ± 0.51	0.44 ± 0.01	58.05 ± 0.84	0.88 ± 0.08	2.88 ± 0.03
NADES9 ^c,d,e,h,j,k,m^	189.5 ± 8.5	2.18 ± 0.10	304.9 ± 6.3	92.8 ± 1.4	0.75 ± 0.12	0.21 ± 0.06	81.8 ± 1.3	0.55 ± 0.02	3.03 ± 0.07
MeOH ^m^	459.3 ± 9.3	3.69 ± 0.09	690.8 ± 9.7	212.6 ± 4.3	3.33 ± 0.45	0.63 ± 0.09	34.98 ± 0.58	0.89 ± 0.04	2.83 ± 0.08

* Means of triplicate analyses ± standard deviation; Values in the rows followed by different letters are significantly different at *p* < 0.05 according to paired *t*-test.

## Data Availability

All data are included in the main text.

## Supplementary Materials

The following supporting information can be downloaded at: https://www.mdpi.com/article/10.3390/plants11182346/s1, Table S1: Influence of water content on TPC, TFC, and RSA values of *A. eupatoria* obtained using three different NADES (mean ± standard deviation from duplicate); Table S2: Influence of water content on phenolic compositions of *A. eupatoria* obtained from three different NADESs (expressed as mg/kg of dried sample); Table S3: TPC, TFC, and RSA of *A. eupatoria* obtained using NADES and methanol (mean ± standard deviation from duplicate); Table S4: Paired *t*-test; Table S5: The β∞ values for 5 phenolic compounds from *A. eupatoria* in nine NADESs, methanol, and water; Table S6: The $\beta_{\mathrm{NADES}}^{\infty }/\beta_{\mathrm{water}}^{\infty }$ values for 5 phenolic compounds in nine NADESs. The values show the relative extractability of a solute in NADES compared with pure water; Table S7: The logP values for the five most abundant phenolic compounds in *A. eupatoria* extract predicted by COSMO-RS; Figure S1: The sigma profiles of rutin and NADES 2 (choline chloride: tartaric acid 1:1). The surface charge density of rutin is shown in the upper-left part of the graph; Figure S2: The sigma profiles of isoquercetin and NADES 5 (choline chloride: glycerol 1:1). The surface charge density of isoquercetin is shown in the upper-left part of the graph.
