# Supplementary material for: Efficiency of Natural Deep Eutectic Solvents to Extract Phenolic Compounds from Agrimonia eupatoria: Experimental Study and In Silico Modelling
Source: Plants (Basel). 2022 Sep 8;11(18):2346. doi: 10.3390/plants11182346 (PMC9501009; doi:10.3390/plants11182346)
Supplement: Supplementary file 1 [file plants-11-02346-s001.zip › plants-1890160-supplementary.pdf]

Efficiency of natural deep eutectic solvents to extract flavonol glycosides from *Agrimonia eupatoria*: experimental study supported by COSMO-RS

Mila Lazović[1*], Ilija Cvijetić[2*], Milica Jankov[1], Dušanka Milojković-Opsenica[2], Jelena Trifković[2], Petar Ristivojević[2]**

[1] *Innovation Centre of Faculty of Chemistry Ltd, Studentski trg 12-16, 11158 Belgrade, Serbia*
[2] *University of Belgrade – Faculty of Chemistry, Studentski trg 12-16, 11158 Belgrade, Serbia*
* Equally contributed
** Correspondence: jvelicko@chem.bg.ac.rs

Supplementary materials

List of tables and figures:

Table S1 Influence of water content TPC, TFC and RSA values of A. eupatoria obtained using three different NADES (mean ± standard deviation from duplicate).

Table S2 Influence of water content on phenolic compositions of A. eupatoria obtained from three different NADESs (expressed as mg/kg of dried sample).

Table S3 TPC, TFC and RSA of A. eupatoria obtained using NADES and methanol (mean ± standard deviation from duplicate).

Table S4 Paired t-test.

Table S5 The β∞ values for 5 phenolic compounds from *A. eupatoria* in nine NADES solvents, methanol and water.

Table S6 The $\beta^{\infty}_{\mathrm{NADES}}/\beta^{\infty}_{\mathrm{water}}$ values for 5 phenolic compounds in nine NADES solvents. The values show relative extractability of a solute in NADES solvent compared with pure water.

Table S7 The logP values for five most abundant phenolic compounds in *A. eupatoria* extract predicted by COSMO-RS.

Figure S1 The sigma profiles of rutin and NADES 2 (choline chloride: tartaric acid 1:1). The surface charge density of rutin is shown in the upper left part of the graph.

Figure S2 The sigma profiles of isoquercetin and NADES 5 (choline chloride: glycerol 1:1). The surface charge density of isoquercetin is shown in the upper left part of the graph.

Table S1 Influence of water content TPC, TFC and RSA values of *A. eupatoria* obtained using three different NADES (mean ± standard deviation from duplicate).

water content % w/w	RSA mmol TE/kg	TPC g GAE/kg	TFC g RUE/kg
	Choline chloride: Tartaric acid 1:1		
20%	260.1 ± 1.9	25.74 ± 0.59	38.1 ± 1.1
30%	258 ± 23	26.56 ± 0.99	39.5 ± 5.0
40%	275 ± 21	26.52 ± 0.94	45.5 ± 0.4
50%	351 ± 23	34.3 ± 1.1	50.9 ± 6.5
	Choline chloride: Urea 1:2		
20%	395 ± 20	40.00 ± 0.86	67.79 ± 0.86
30%	376 ± 22	37.17 ± 0.96	52.3 ± 6.0
40%	445 ± 22	43.00 ± 0.56	51.60 ± 0.26
50%	317 ± 2	32.66 ± 0.60	39.3 ± 2.3
	Choline chloride: Succinic acid 1:1		
20%	196.6 ± 4.3	19.64 ± 0.11	22.2 ± 1.8
30%	107.7 ± 9.2	9.96 ± 0.22	13.52 ± 0.42
40%	479.6 ± 9.4	44.2 ± 1.1	55.9 ± 3.0
50%	330 ± 23	31.4 ± 1.4	45.3 ± 9.2

Table S2 Influence of water content on phenolic compositions of *A. eupatoria* obtained from three different NADESs (expressed as mg/kg of dried sample).

water content (%w/w)	Protocatechuic acid	Syringic acid	Chlorogenic acid	p-Hydroxybenzoic acid	Aesculetin	Caffeic acid	Isoorientin	Rutin	Vitexin	Isoquercetin	p-Coumaric acid	Quercitrin	Astragalin	Rosmarinic acid	Luteolin	Quercetin	Naringenin	Kaempferol
	Choline chloride: Tartaric acid 1:1																	
20%	10.09	12.39	11.32	10.87	1.91	2.81	0.75	64.57	15.87	316.3	4.04	460.9	152.1	1.92	0.43	170.5	1.62	5.92
30%	8.10	8.87	17.27	18.48	3.27	5.43	1.38	119.41	23.69	421.6	4.60	596.8	212.6	3.08	0.76	316.1	2.57	11.37
40%	5.85	8.06	15.86	10.53	3.29	5.04	1.12	112.36	22.67	433.1	3.80	618.6	220.1	3.45	0.65	62.8	1.03	3.13
50%	5.79	6.64	21.89	9.54	4.83	4.17	1.30	134.17	28.33	494.5	4.20	708.2	272.5	4.20	0.62	43.1	0.75	2.38
	Choline chloride: Urea 1:2																	
20%	6.72	15.21	43.74	14.81	NF	3.48	1.53	146.5	36.13	522.9	9.44	746.3	314.5	3.65	0.94	11.65	1.95	4.55
30%	4.55	7.18	29.91	19.25	1.70	1.82	1.66	130.3	28.49	496.3	7.01	693.8	264.6	2.85	0.86	15.84	1.35	3.35
40%	5.16	4.91	31.48	22.54	6.71	6.03	2.00	139.3	31.47	538.6	10.88	761.2	323.5	4.97	1.35	33.09	1.30	4.12
50%	6.62	11.91	40.57	30.54	7.12	5.62	1.53	129.6	27.87	491.8	11.48	681.8	269.7	4.19	1.03	13.98	1.37	3.20
	Choline chloride: Succinic acid 1:1																	
20%	3.96	2.52	9.21	10.57	1.39	2.19	1.07	54.20	14.55	338.1	2.88	469.8	163.3	1.26	0.38	20.96	0.45	2.26
30%	2.70	3.66	2.77	4.11	1.09	1.81	0.25	22.90	5.10	163.3	0.80	210.6	74.7	0.63	0.15	16.10	0.25	2.12
40%	4.82	3.11	18.21	17.41	3.86	5.12	1.74	163.05	33.12	548.4	8.56	846.5	340.1	4.44	1.06	67.85	1.55	6.13
50%	4.21	5.35	17.41	25.63	2.70	3.77	1.04	110.62	23.02	471.9	4.46	675.9	254.2	2.66	0.59	35.11	0.85	2.44

Table S3 TPC, TFC and RSA of *A. eupatoria* obtained using NADES and methanol (mean ± standard deviation from duplicate).

Solvents	RSA mmol TE/kg	TPC g GAE/kg	TFC g RUE/kg
NADES1	263.4 ± 8.7	38.83 ± 0.57	54.9 ± 2.1
NADES2	260.1 ± 1.9	25.74 ± 0.59	38.1 ± 1.1
NADES3	397 ± 20	40.00 ± 0.86	67.79 ± 0.86
NADES4	196.6 ± 4.3	19.64 ± 0.11	22.2 ± 1.8
NADES5	413 ± 35	41.6 ± 1.1	57.2 ± 4.2
NADES6	446 ± 37	39.10 ± 0.66	53.7 ± 2.1
NADES7	296 ± 14	24.46 ± 0.14	35.4 ± 2.6
NADES8	270 ± 11	33.20 ± 0.59	44.8 ± 3.7
NADES9	75.6 ± 4.3	8.32 ± 0.19	10.69 ± 0.18
MeOH	416.0 ± 0.6	35.73 ± 0.56	60.4 ± 5.2

Table S4 Paired *t*-test.

	t^*	*P*
MeOH vs NADES1	1.10	0.29
MeOH vs NADES2	1.09	0.29
MeOH vs NADES3	2.21	0.04
MeOH vs NADES4	2.01	0.06
MeOH vs NADES5	2.22	0.04
MeOH vs NADES6	1.43	0.17
MeOH vs NADES7	0.33	0.74
MeOH vs NADES8	1.29	0.22
MeOH vs NADES9	1.82	0.09
NADES1 vs NADES2	2.31	0.03
NADES1 vs NADES3	0.48	0.64
NADES1 vs NADES4	2.67	0.02
NADES1 vs NADES5	1.35	0.19
NADES1 vs NADES6	0.44	0.67
NADES1 vs NADES7	1.21	0.24
NADES1 vs NADES8	0.82	0.42
NADES1 vs NADES9	2.45	0.03
NADES2 vs NADES3	1.55	0.14
NADES2 vs NADES4	0.95	0.36
NADES2 vs NADES5	1.76	0.10
NADES2 vs NADES6	1.24	0.23
NADES2 vs NADES7	1.06	0.31
NADES2 vs NADES8	1.34	0.20
NADES2 vs NADES9	2.49	0.02
NADES3 vs NADES4	2.30	0.03
NADES3 vs NADES5	1.96	0.07
NADES3 vs NADES6	2.56	0.02
NADES3 vs NADES7	2.67	0.02
NADES3 vs NADES8	1.73	0.10
NADES3 vs NADES9	2.02	0.06
NADES4 vs NADES5	2.19	0.04
NADES4 vs NADES6	2.04	0.06
NADES4 vs NADES7	2.01	0.06
NADES4 vs NADES8	2.23	0.04
NADES4 vs NADES9	1.54	2.11
NADES5 vs NADES6	2.30	0.03
NADES5 vs NADES7	2.31	0.03
NADES5 vs NADES8	2.07	0.05
NADES5 vs NADES9	2.03	0.06
NADES6 vs NADES7	2.03	0.06
NADES6 vs NADES8	0.44	0.67
NADES6 vs NADES9	1.84	0.08
NADES7 vs NADES8	1.46	0.16
NADES7 vs NADES9	1.81	0.09
NADES8 vs NADES9	1.97	0.06

$^* t_{cr} = 2.11$

Table S5 The β∞ values for 5 phenolic compounds from *A. eupatoria* in nine NADES solvents, methanol and water.

	Pro:MA (1)	ChCl:TA (2)	ChCl:Urea (3)	ChCl:SA (4)	ChCl:Gl (5)	Gl:Urea (6)	Gl:Urea 2:1 (7)	Gl:LA (8)	Gl:AA (9)	MeOH	H_2O (×10^{-3})
Isoquercetin	8.9	24.0	3.2	5.7	21.0	1.4	4.0	4.1	1.5	82.1	0.96
Rutin	111	400	1.6	18.8	11.3	0.5	1.6	2.5	1.6	50.9	0.16
Quercitrin	5.4	9.4	2.4	4.1	54.7	1.0	3.2	3.8	1.3	92.7	0.31
Astragalin	15.8	36.6	1.4	7.3	23.9	0.5	1.4	2.1	1.2	26.0	0.39
Quercetin	2.4	2.7	124	11.8	983	83.4	298	179	20.8	7527	27

Table S6 The $\beta^{\infty}_{NADES}/\beta^{\infty}_{water}$ values for 5 phenolic compounds in nine NADES solvents. The values show relative extractability of a solute in NADES solvent compared with pure water.

	L-Pro:MA (1)	ChCl:TA (2)	ChCl:Urea (3)	ChCl:SA (4)	ChCl:Gl (5)	Gl:Urea (6)	Gl:Urea 2:1 (7)	Gl:LA (8)	Gl:AA (9)
Isoquercetin	9333	25064	3370	60101	22015	1497	4204	4286	1533
Rutin	680110	2453642	10084	115244	69056	3297	9531	15421	9856
Quercitrin	17509	30805	7754	13388	178288	3306	10391	12365	4134
Astragalin	40223	93092	3648	18655	60960	1338	3522	5272	2940
Quercetin	91.1	99.3	4623	442.0	36721	3116	11125	6721	777.7

Table S7 The logP values for five most abundant phenolic compounds in *A. eupatoria* extract predicted by COSMO-RS.

**Compound**	**log*P***
Isoquercetin	1.877
Rutin	1.925
Quercitrin	2.766
Astragalin	2.108
Quercetin	3.191

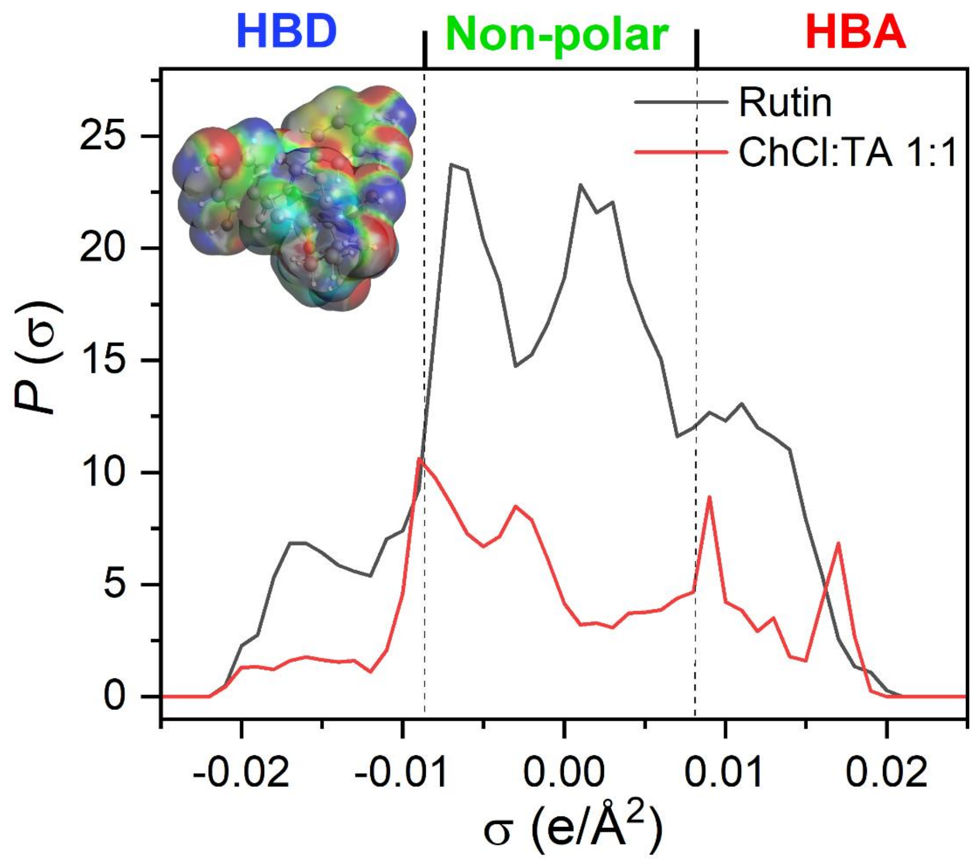

Figure S1 The sigma profiles of rutin and NADES 2 (choline chloride: tartaric acid 1:1). The surface charge density of rutin is shown in the upper left part of the graph.

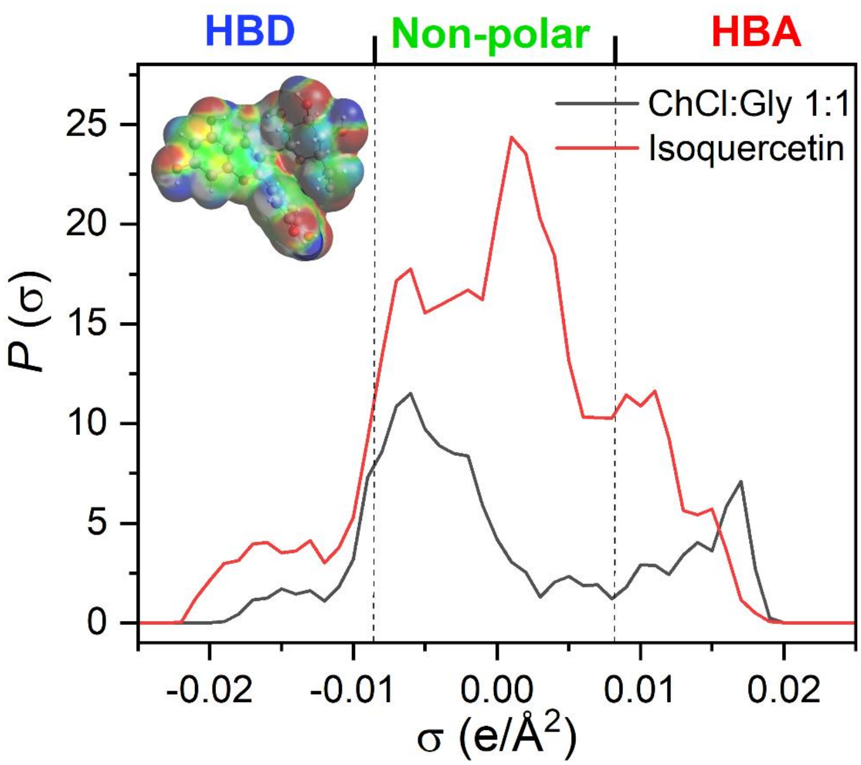

Figure S2 The sigma profiles of isoquercetin and NADES 5 (choline chloride: glycerol 1:1). The surface charge density of isoquercetin is shown in the upper left part of the graph.
